# Novel patient-reported outcomes (PROs) used in a pilot and feasibility study of a Cognitive Behavioral Coping Skills (CBCS) group intervention for patients with chronic hepatitis C

**DOI:** 10.1186/s40814-018-0285-5

**Published:** 2018-06-27

**Authors:** Donna M. Evon, Carol E. Golin, Rachel Ruffin, Shauna Ayres, Michael W. Fried

**Affiliations:** 1 Division of Gastroenterology and Hepatology, University of North Carolina, CB# 7584, 8010 Burnett-Womack, Chapel Hill, NC 27599 USA; 2 Department of Medicine, University of North Carolina, Chapel Hill, NC USA; 3 Department of Health Behavior, University of North Carolina, Chapel Hill, NC USA; 4 Durham VA Medical Center, Durham, NC USA

**Keywords:** Liver disease, Patient-reported outcome measures, Antiviral therapy, Direct-acting antiviral, Psychological, Quality of life, Symptoms, Stress

## Abstract

**Background:**

Patients with chronic hepatitis C virus (HCV) experience reduced quality of life, HCV-associated symptoms, comorbid conditions, and treatment side effects. The Cognitive Behavioral Coping Skills group intervention for HCV (CBCS-HCV) was developed using the Stage Model of Behavioral Therapies Research. Intervention development and initial feasibility testing in wave 1 participants were previously reported. The primary objective of this subsequent pilot with wave 2–3 participants was to investigate the effect sizes and clinical improvements in patient-reported outcomes (PROs) and trial and intervention feasibility.

**Methods:**

A pilot feasibility two-arm randomized controlled trial using block randomization to assign patients to CBCS-HCV or standard of care was conducted. Participants attended nine group sessions: four before HCV treatment and five during treatment. PRO data were collected at five time points: before the CBCS intervention (T1), immediately before HCV treatment (T2), during HCV treatment (T3, T4), and 1 month post-intervention/post-HCV treatment (T5). PROs included quality of life, perceived stress, HCV symptoms, and medication adherence. Cohen’s *d* was used to estimate within-group changes (WGCs) and between-group differences (BGDs), with *d* > 0.35 considered potentially clinically significant. Potential mechanisms of change were also evaluated.

**Results:**

Several WGCs and BGDs (ES > .35) suggest that the CBCS-HCV may promote improvements in PROs: psychological stress, depression, anger, anxiety, sleep disturbance, and fatigue. The intervention did not appear to impact social functioning, pain, or medication adherence. Cognitive behavioral skills and group therapy dynamics, but not HCV treatment self-efficacy, may mediate improvements in PROs. Most aspects of the study trial, including intervention implementation, were feasible. Patient acceptance and retention were exceptional. The greatest feasibility challenge was due to patients needing to initiate treatment as soon as medications were obtained, but often before a full block could be created in wave 3. Challenges with PRO data collection were identified that will be resolved in future studies.

**Conclusions:**

The CBCS-HCV intervention warrants future investigation in an efficacy trial to evaluate improvements in selected PROs. The next step is to pilot test the CBCS-HCV delivered via telehealth to an expanded pool of patients to reduce patient barriers, hone technical logistics, and improve intervention reach and effectiveness.

**Trial registration:**

NCT03057236 Retrospectively registered.

## Background

Chronic hepatitis C virus (HCV) affects over three million people in the USA and can lead to advanced liver disease, liver-related mortality, and all-cause mortality [1, 2]. Patients complain of several diffuse symptoms that may be associated with HCV and report poor health-related quality of life (HRQOL) [3, 4]. Antiviral treatment can eradicate HCV but can be difficult to tolerate for some patients [5]. Despite the availability of more tolerable and effective treatments, there remains a substantial need to develop psychosocial interventions to improve the health and well-being of the underserved HCV population [6]. There is also a need in clinical hepatology studies to capture patient-reported experiences using patient-reported outcome measures (PROMs) to complement traditional clinician-reported adverse events and laboratory markers [7, 8]. Psychosocial interventions could enhance patients’ coping skills to mitigate the negative impact of disease, and treatment in patients have difficulty coping with treatment side effects. Additionally, newer regimens are allowing more individuals to undergo treatment (i.e. traditionally “difficult to treat” patients with psychiatric or addiction issues); however, this introduces new clinical challenges for treatment providers. Psychosocial interventions provided before or during HCV treatment could provide patients with ancillary support and skills training that medical providers do not have the time nor expertise to provide [9, 10]. Finally, the current repertoire of healthcare services available for HCV patients is woefully limited. Many patients do not meet the eligibility requirements for insurance approvals of HCV treatment and are left with no other healthcare alternatives to enhance overall health and well-being [11]. Therefore, psychosocial interventions could be useful for patients awaiting treatment, undergoing treatment, or those who have completed treatment but are still living with advanced liver disease or cirrhosis. Psychosocial interventions that assist patients in developing cognitive and behavioral coping skills have been around for decades. PROMs are often the best method for evaluating clinical improvements in mental and physical health outcomes in other medical populations but are used less often in clinical hepatology studies [8].

To address the gap in healthcare services for HCV patients, we developed the Cognitive Behavioral Coping Skills group intervention for HCV (CBCS-HCV), an intervention that was modified specifically for the needs of HCV patients undergoing antiviral therapy. Several novel PROMs were used to evaluate improvements in functioning, stress, and symptoms which have not previously been utilized in hepatology studies. The formative work, preliminary study protocol, and initial pilot feasibility testing of the CBCS-HCV in an initial wave of participants (“wave 1”) undergoing interferon-based therapy have been previously described [12]. As discussed in the prior article, we followed the Stage Model for Behavioral Therapies Research guide to develop the CBCS-HCV [13]. In the initial phase of this research program, we addressed several essential steps recommended in stage 1a and stage 1b research. First, we performed several research activities, such as a literature review, a patients’ needs assessment, and selected pre-existing intervention materials to modify for the CBCS-HCV [12]. Next, we developed the CBCS-HCV group intervention Patient Workbook and Therapist Guide and a preliminary study protocol to conduct a mini randomized controlled trial (RCT). Several PROs were selected to evaluate a broad array of potential outcomes that we speculated might be affected by the intervention. Finally, we conducted initial feasibility and pilot testing in wave 1 study participants to evaluate (a) patient acceptability; (b) feasibility of recruitment, randomization, retention, and PRO data collection; (c) feasibility of intervention delivery; and (d) therapist protocol fidelity [12].

Several lessons were learned during the initial pilot testing. Patient acceptability was exceptional, therapist protocol fidelity was high, and participation, retention, and data collection were successful [12]. The major challenge was enrollment impediments that prevented block randomization due to providers and patients deferring HCV treatment until newer treatment regimens were available. These challenges were viewed as temporary and unlikely to impede future piloting. Because patient acceptability of the CBCS-HCV was high and the majority of other study and intervention methods were found feasible, we elected to move forward with additional pilot testing with wave 2 and wave 3 study participants to address two additional essential research activities recommended by the Stage Model of Behavioral Therapies prior to conducting a full efficacy study [12].

We placed emphasis on selecting and evaluating novel PROs not often utilized in clinical hepatology studies, although objective markers (viral load, pill count) were collected as well. Many clinical hepatology studies are based on clinician-reported adverse events or improvements, which do not correlate well with patient-reported harms or improvements [14, 15]. Since the focus of this pilot study was a psychological/behavioral intervention, it was critical to capture clinical improvements from the patients’ perspective. Selection of PROs was partially based on the Wilson and Cleary classification scheme [16]. The model suggests that to be very precise and valid, distinctly different PROs should be assessed separately to maximize information and decrease overlap between constructs. Symptoms and side effects are the most proximally and least confounded clinical variables associated with disease and treatment; however, as one moves further away from the direct effects of disease and treatment, towards the broader construct of quality of life, many other confounding patient and environmental factors influence a quality of life score. Therefore, we selected several PROMs that captured unidimensional patient-reported symptoms (e.g., fatigue, depression, sleep, pain) most closely related to HCV and least confounded by other influences. This would allow us to determine within the pilot study, the precise outcomes that could be affected by the intervention.

The primary objectives of this follow-up pilot feasibility study in wave 2 and 3 study participants were as follows: (1) to examine effect size (ES) estimates of change in specific PROs, (2) to determine whether clinically significant improvements occurred in any specific PROs, and (3) to continue to evaluate study feasibility elements to inform a larger efficacy study. This information can be used to inform the selection of PROs and sample size calculations for a future efficacy trial. To achieve our first objective, we calculated ES estimates for all potential PROs to examine within-group changes (WGCs) over time and between-group differences (BGDs) at various time points. To achieve our second objective, we focused on ES estimates from baseline (T1) to the start of HCV treatment (T2), which aligned with receipt of four out of the nine CBCS-HCV sessions to determine the impact of half of the intervention on PROs prior to HCV treatment initiation. We also focused on ES estimates from baseline (T1) to 1 month post-CBCS-HCV intervention/post-HCV treatment (T5) to examine sustained clinical improvements post-intervention/post-treatment. We did not anticipate improvements in PROs during HCV treatment in the treatment condition because treatment side effects could lead to worse PRO scores, but we speculated that symptoms might either stabilize, or not worsen, during treatment. We selected an ES estimate of *d* > 0.35 as the threshold to define a potentially clinically significant improvement in a PRO, as it represented a small to moderate effect size and would be worthy of testing in a future efficacy trial. Study feasibility elements evaluated to address the third objective included feasibility of a RCT study design, intervention delivery, patient acceptability, therapist protocol fidelity, recruitment, enrollment, attendance, retention, and PRO data collection.

## Methods

### Study design

A detailed description of the preliminary study design and methods for an RCT of the CBCS-HCV intervention with wave 1 participants has been published [12]. Only a brief description of the study protocol conducted with wave 2 and 3 study participants is provided below. To test the feasibility of conducting a future RCT, the pilot feasibility study was designed as a two-arm RCT with study participants randomized to CBCS-HCV (*n* = 12) or standard of care (SC; *n* = 12). We sought to randomize patients for wave 2 when a block of 12 patients were consented and to repeat this procedure for wave 3 when a second block of 12 patients were consented. In this paper, we briefly describe the updated version of the study protocol, feasibility of implementation, and PRO results in wave 2 and wave 3 pilot testing for further refinement to prepare for a larger efficacy study.

### Inclusion/exclusion criteria

We applied the same criteria that were used during wave 1 piloting to the current study, with the exception of two changes. Only patients prescribed a 12-week HCV regimen were eligible (i.e., patients prescribed 24-week regimens were excluded) so that all patients were maintained on the same intervention and PRO assessment schedule during and after the intervention. Secondly, patients co-infected with HIV or hepatitis B were included to increase the pool of eligible patients and mirror the type of patients seen in clinical practice.

### Screening and recruitment

Recruitment of 12 patients to enroll in wave 2 occurred in March 2014. Recruitment of 12 patients to enroll in wave 3 occurred from the end of April to the beginning of July in 2014. Data collection was completed in December 2014. Potentially eligible patients were pre-screened from a treatment waitlist, and those potentially eligible were contacted to determine eligibility and interest. Screening and recruitment were conducted by two trained research coordinators. Interested patients attended a baseline research visit after participating in the informed consent process.

### Randomization

A biostatistician developed a computer-based randomization procedure to conduct randomization. The protocol dictated that when a group of 12 eligible patients were consented for wave 2, study participants would be randomized to CBCS-HCV (*n* = 6) and SC (*n* = 6) using a block randomization procedure. The procedure was then repeated to randomize and enroll 12 patients for wave 3. Enrolled participants were contacted later by a research coordinator and informed of group assignment.

### Standard of care (SC) condition

Participants randomized to SC were able to proceed with initiating HCV treatment per standard clinical procedures and were followed and managed by the liver clinicians. At the time of recruitment for wave 2–3 in 2014, two first-generation direct-acting antiviral (DAA) therapies were being used in standard clinical care: simeprevir/sofosbuvir and sofosbuvir/ribavirin [17]. All study subjects were prescribed one of these two regimens. The first regimen involved two pills dosed once per day, while the second regimen contained 7 pills and was dosed half in the morning and half at bedtime. Standard clinical procedures encouraged patients to attend regular follow-up treatment visits to monitor safety and efficacy at treatment weeks 2, 4, 6, 8, and 12, but all of these visits were not required if patients were doing well. Data collection of paper and pencil PROMs was married to these clinic visits. Standard labs were drawn according to clinical protocol and not for research purposes.

### CBCS-HCV intervention condition

The content of the CBCS-HCV intervention was the same as that delivered in wave 1 with two exceptions. Updates were made, as needed, to certain sections (e.g., description of new medications). Secondly, feedback received after wave 1 piloting favored extending the length of each weekly session and adding additional sessions to allow for more group discussion and practice. However, due to insurance restrictions, the clinical team needed patients to start HCV treatment within 1–2 weeks of obtaining their medications or else risk having future refills denied. Due to this restrictive timeline, we needed to condense the CBCS-HCV modules into nine, 2-h sessions: four weekly sessions prior to starting HCV treatment and five sessions delivered at HCV treatment weeks 2, 4, 6, 8, and 12. A description of the condensed modules covered in 9 sessions is provided in Table 1. The study therapist was the same PhD-level licensed clinical psychologist who facilitated the group during wave 1 pilot testing.

**Table 1:** Content of nine CBCS-HCV group modules

Module	Relaxation training	Review and application of previous skills	Training in new topic and skills
1	Progressive muscle relaxation (PMR)	• Introductions • Group expectations	• Intro and overview • Positive lifestyle changes
2	Diaphragmatic breathing	• Positive lifestyle changes	• Stress awareness and appraisal • Lifestyle changes
3	Autogenic training	• Stress and appraisal • Lifestyle changes	• Negative automatic thoughts • Cognitive distortions
4	Healing wellness imagery	• Negative automatic thoughts • Cognitive distortions	• Cognitive restructuring
5	Light imagery	• Cognitive restructuring	• Coping with stress and symptoms
6	Passive PMR	• Coping with stress and symptoms	• Cognitive-behavior skills for depression • Behavioral activation • Pleasurable activities
7	Immune system guided imagery	• Cognitive-behavioral skills for depression • Behavioral activation • Pleasurable activities	• Activity-rest cycles • Sleep hygiene
8	Self-forgiveness script	• Activity-rest cycles • Sleep hygiene	• Anger prevention/management • Interpersonal effectiveness
9	Mindfulness	• Anger prevention/management • Interpersonal effectiveness	• Assertive communication • Interpersonal effectiveness

### PROM data collection schedule

Based on experiences during wave 1 pilot testing, we altered the assessment schedule to capture the PROs for waves 2 and 3. In the current pilot study, we wanted to capture PROs more frequently during the CBCS intervention; therefore, we added an additional PRO assessment after session #8 of the CBCS (aligned with treatment week 8). It was also important to capture post-intervention PRO data closer to the end of the intervention; therefore, we moved the post-intervention PRO assessment from 3 months to 1 month post-intervention. The PRO assessment schedule was as follows:

- T1: baseline PRO assessment after consent in both conditions
- T2: 1–2 weeks before starting HCV treatment (aligned with after session #4 in the CBCS condition)
- T3: at treatment week 8 (aligned with after session #8 in the CBCS condition)
- T4: at the end of HCV treatment at week 12 (aligned with after final session #9 in the CBCS condition)
- T5: at 1-month post-intervention/post-HCV treatment.

Based on negative experiences during wave 1 using electronic monitoring pill caps to objectively measure medication adherence, we decided to measure adherence using only pill counts and patient self-report. Electronic monitoring caps were eliminated. Medication adherence (pill counts, self-report) was evaluated at every clinic visit attended during treatment (weeks 2, 4, 6, 8, and 12).

### Participant reimbursement

Participants in each condition were compensated $25 for completion of each of the five PRO assessments T1–T5. For wave 2, CBCS-HCV participants were compensated $25 for attendance at each of the 9 group sessions. To enhance recruitment for wave 3, participants assigned to the CBCS-HCV intervention were reimbursed $100 to attend each of the first four pre-treatment CBCS sessions to help defray travel cost for research only, non-clinical visits.

### Measures

#### PROMs

See detailed description of the PROMs in our previous article [12]. PROMs were collected via paper and pencil, typically after regular clinical visits and sometimes via post mail if clinical visits did not align with the PRO assessment schedule. Generic health-related quality of life (HRQOL) was measured using the Functional Assessment of Cancer Therapy-General Population (FACT-GP) instrument [18]. The FACT-GP instrument has a total score and four subscales: physical well-being, social/family well-being, emotional well-being, and functional well-being. Perceived stress was measured using the Perceived Stress Scale (PSS) [19]. Precise HCV symptoms and treatment side effects were measured using eight short instruments from the National Institutes of Health (NIH) Patient-Reported Outcomes Measurement Information System® (PROMIS®): depression—8; irritability—8; anxiety—4; fatigue—8; sleep disturbance—8; sleep-related impairment—8; pain intensity—3; and pain interference—4 [20, 21]. Medication adherence was measured at each treatment clinic visit using pill counts and self-report, per protocol described previously [12]. Pill count was conducted for all oral medications. Medical records were reviewed for laboratory data, specifically HCV RNA viral load at 4 or 12 weeks post-HCV treatment to determine if the virus was detectable or undetectable.

#### Process measures

See description in previous article [12]. Because it will be important to measure potential mechanisms by which clinical gains are made in a future efficacy study, we piloted three PRO surveys that could potentially capture putative mechanisms: active CBCS skills (e.g., relaxation, awareness of tension, assertiveness, coping confidence) using the Measure of Current Status (MOCS) [22], HCV treatment self-efficacy [23], and nonspecific therapeutic aspects of group therapy [24–26].

#### Study feasibility measures

Similar to the protocol implemented in wave 1 pilot testing, we evaluated the feasibility of the following study elements: (a) feasibility of randomization based on the ability to randomize and enroll a block of 12 participants; (b) recruitment and enrollment efforts as evidenced by the proportion of patients screened, consented, and enrolled; (c) retention efforts based on the number of CBCS-HCV sessions attended and the proportion of patients who started and completed the CBCS-HCV intervention; and (e) the feasibility of data collection by the average of data points completed at each assessment period.

#### Therapist protocol fidelity measures

##### Therapist’s adherence and competency with delivery of CBCS-HCV modules

Details of this measure are described elsewhere [12]. Study staff observed the delivery of the CBCS-HCV using a 0–100% rating scale to track the proportion of each module subsection that was completed according to the protocol. Staff also rated the therapist’s competency during each session using a 14-item scale.

##### Participant acceptability and comprehension scale

Details of this measure are described elsewhere [12]. CBCS participants completed a 14-item survey rating each session on acceptability.

### Data analysis

Data were analyzed in IBM SPSS software v.23 and SAS (Cary, NC). Descriptive statistics (means, medians, standard deviations) were calculated for continuous variables. Total scores and/or subscale scores of each PROM were calculated per instrument instructions. Higher scores on the FACT-GP instrument indicate better quality of life. Higher scores on the PSS indicate higher perceived stress. A publicly available PROMIS® scoring system was used to sum total raw scores and translate the raw scores into standardized *T*-scores, which have been calibrated in the US general population to have a mean of 50 and a standard deviation (SD) of 10. Thus, a *T*-score of 40 is one SD below, and a *T*-score of 60 is one SD above, the US general population mean. For all of the PROMIS® instruments, a higher *T*-score indicates a higher degree of that construct (e.g., higher depression, higher pain intensity).

Tests of statistical significance were not conducted as this was a pilot study. The magnitude of difference between two PRO means were calculated using Cohen’s *d* as an estimate of effect size [27]. Cohen’s *d* was used to indicate the standardized difference between two PRO means within the same condition over time (i.e., within-group change (WGC)) and between two conditions at the same time point (i.e., between-group differences (BGDs)). To determine the difference between two PRO means, Cohen’s *d* is defined as the difference between the two means divided by the *pooled* standard deviation ((*M*_2_ − *M*_1_)/SD_pooled_). We applied Cohen’s interpretation of ES estimates as follows: (a) a large ES or magnitude of difference = .80 or 8/10 of a standard deviation, (b) a moderate ES or magnitude of difference = .50 or 1/2 of a standard deviation, and (c) a small ES or magnitude of difference = .20 or 1/5 of a standard deviation. ES estimates were calculated for all key PROs to determine the magnitude of WGC (e.g., the change in depression mean scores in the CBCS condition from T1 to T2) and BGD (e.g., depression mean scores in the CBCS and SC conditions at T2). We focused on potential clinical improvements (defined as ES > .35) from T1 to T2 in the CBCS-HCV condition to interpret the impact of having received half of the CBCS intervention sessions, in particular those delivered prior to initiation of HCV treatment, and from T1 to T5 to evaluate any lasting improvements post-intervention/post-HCV treatment.

## Results

### Baseline patient characteristics

Characteristics of study participants are displayed in Table 2.

**Table 2:** Patient characteristics

		CBCS (*n* = 9)	SOC (*n* = 11)	Total (*n* = 20)
		*N* (%)	*N* (%)	*N* (%)
Condition	Wave 2	5 (56%)	6 (54%)	11 (55%)
	Wave 3	4(44%)	5 (46%)	9 (45%)
Age (years)	Mean (range)	63 (56–73)	58 (32–72)	60 (32–73)
Sex	Male	5 (56%)	7 (64%)	12 (60%)
	Female	4 (44%)	4 (36%)	8 (40%)
Race	White	5 (56%)	8 (73%)	13 (65%)
	Black	4 (44%)	2 (18%)	6 (30%)
	Other	0 (0%)	1 (9%)	1 (5%)
Marital status	Single	3 (33%)	43 (36%)	7 (35%)
	Married or living with partner	3 (33%)	5 (46%)	8 (40%)
	Separated, divorced, or widowed	3 (33%)	2 (18%)	5 (25%)
Education level	< 4 year college	4 (44%)	9 (82%)	13 (65%)
	≥ 4 year college	5 (56%)	2 (18%)	7 (35%)
Insurance type	Private	1 (11%)	6 (55%)	7 (35%)
	Medicaid/Medicare	7 (78%)	4 (36%)	11 (55%)
	Uninsured	1 (11%)	1 (9%)	2 (10%)
HCV genotype	Genotype 1	7 (78%)	6 (55%)	13 (65%)
	Genotype 2 or 3	2 (22%)	5 (45%)	7 (35%)
HCV treatment	SOF/RBV	2 (22%)	4 (36%)	6 (30%)
	SIM/SOF	7 (78%)	7 (64%)	14 (70%)
Cirrhosis	Yes	2 (22%)	5 (45%)	7 (35%)
	No	7 (78%)	6 (55%)	13 (65%)

### PROMs

**Fig. 1:**
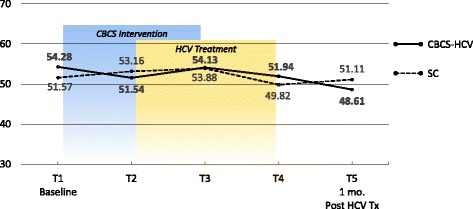
PROMIS depression

**Fig. 2:**
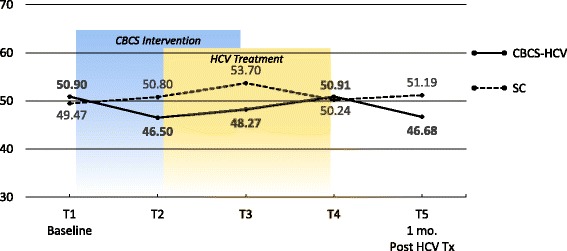
PROMIS anger

**Fig. 3:**
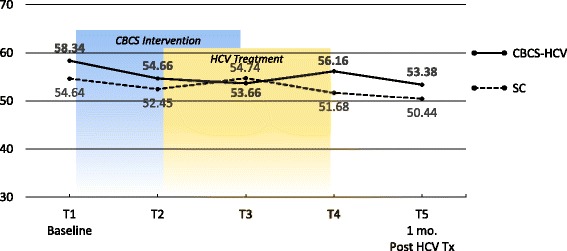
PROMIS anxiety

**Fig. 4:**
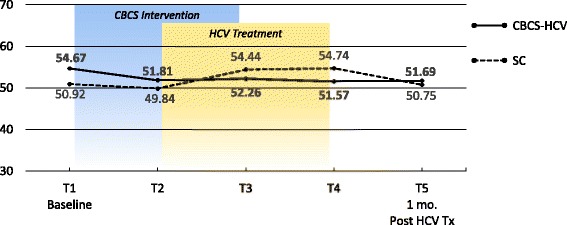
PROMIS sleep disturbance

**Fig. 5:**
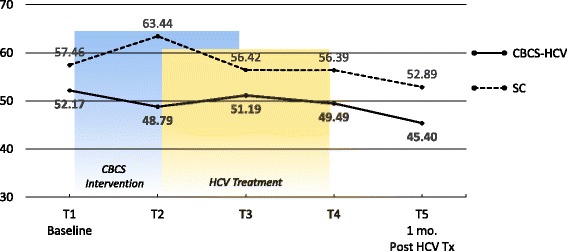
PROMIS sleep-related impairment

**Fig. 6:**
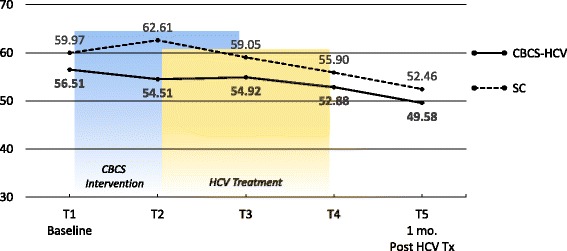
PROMIS fatigue

**Fig. 7:**
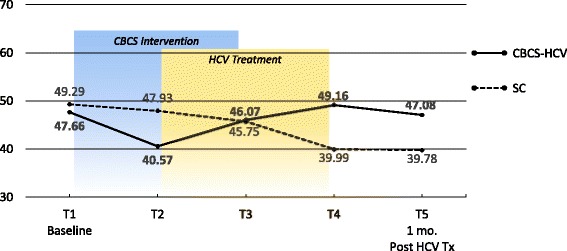
PROMIS pain intensity

**Fig. 8:**
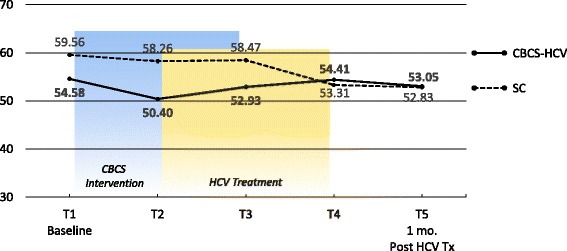
PROMIS pain interference

Means and ESs for all of the PROMs are presented in Tables 3 and 4, respectively. For each variable, the mean and ES for both the CBCS and SC condition across all five time points are provided. Effect sizes connoting WGC from T1 to all subsequent time frames are provided in parentheses after means in Table 4. The third row in Table 4 for each variable provides the ES for BGD of the CBCS and SC at each time point (e.g., ES difference between CBCS and SC at T2). Figures 1, 2, 3, 4, 5, 6, 7, and 8 provide visual graphs for eight PROMIS® measures over time for both conditions.

**Table 3:** Means, effect sizes for within-group changes (WGCs) from T1 within each condition, and effect sizes for between-group differences (BGDs) at each time point

PROMs	Condition	T1 mean	T2 mean	T3 mean	T4 mean	T5 mean
HrQOL total	CBCS	10.54	11.79	10.46	11.54	12.15
	SC	10.06	8.78	10.14	11.61	12.63
HrQOL: physical well-being	CBCS	2.65	3.07	2.83	2.83	3.25
	SC	2.70	2.24	2.70	2.9	3.04
HrQOL: social/family well-being	CBCS	2.79	3.03	2.8	2.96	3.09
	SC	2.82	2.71	2.68	3.10	3.16
HrQOL: emotional well-being	CBCS	2.72	3.00	2.75	3.17	3.13
	SC	2.34	2.50	3.00	3.28	3.25
HrQOL: functional well-being	CBCS	2.38	2.69	2.33	2.57	2.69
	SC	2.20	1.47	1.67	2.33	2.79
Perceived stress	CBCS	1.63	1.27	1.60	1.50	1.29
	SC	1.79	2.09	1.91	1.54	1.33
Depression	CBCS	54.28	51.54	54.13	51.94	48.61
	SC	51.57	53.16	53.88	49.82	51.11
Anger	CBCS	50.90	46.50	48.27	50.91	46.68
	SC	49.47	50.80	53.70	50.24	51.19
Anxiety	CBCS	58.34	54.66	53.66	56.16	53.38
	SC	54.64	52.45	54.74	51.68	50.44
Fatigue	CBCS	56.51	54.51	54.92	52.88	49.58
	SC	59.97	62.61	59.05	55.90	52.46
Sleep disturbance	CBCS	54.67	51.81	52.26	51.57	51.69
	SC	50.92	49.84	54.44	54.74	50.75
Sleep-related impairment	CBCS	52.17	48.79	51.19	49.49	45.40
	SC	57.46	63.44	56.42	56.39	52.89
Pain intensity	CBCS	47.66	40.57	46.07	49.16	47.08
	SC	49.29	47.93	45.75	39.99	39.78
Pain interference	CBCS	54.58	50.40	52.93	54.41	53.05
	SC	59.56	58.26	58.47	53.31	52.83
MOCS total	CBCS	2.25		2.51	2.89	2.92
	SC	2.33		2.31	2.60	2.64
MOCS: relaxation	CBCS	1.78		2.44	2.89	2.69
	SC	1.73		1.65	2.15	2.13
MOCS: awareness of tension	CBCS	2.44		2.78	3.11	2.88
	SC	2.67		2.53	2.77	3.04
MOCS: assertiveness	CBCS	2.44		2.44	2.74	3.25
	SC	2.67		2.70	2.77	2.60
MOCS: coping confidence	CBCS	2.33		2.38	2.82	2.88
	SC	2.27		2.34	2.72	2.79
Tx self-efficacy: patient Comm.	CBCS	9.22		8.41		
	SC	8.79		9.36		
Tx self-efficacy: coping with physical	CBCS	7.36		6.89		
	SC	5.61		6.20		
Tx self-efficacy: coping with Psych.	CBCS	6.87		7.18		
	SC	7.65		6.42

**Table 4:** Effect sizes for within-group changes (WGCs) from T1 within each condition and effect sizes for between-group differences (BGDs) at each time point

PROMs	Condition	T1	T2	T3	T4	T5
			WGC ES^a^	WGC ES^a^	WGC ES^a^	WGC ES^a^
HrQOL: physical well-being	CBCS		.44	.18	.18	.59
	SC		− .47	.00	.20	.37
	(BGD ES^b^)	(− .05)	(.92)	(.15)	(− .06)	(.23)
HrQOL: emotional well-being	CBCS		.33	.03	.52	.54
	SC		.15	.70	1.07	1.00
	(BGD ES^b^)	(.41)	(.51)	(− .29)	(− .14)	(− .19)
HrQOL total	CBCS		.39	− .02	.33	.51
	SC		− .37	.03	.54	.90
	(BGD ES^b^)	(.15)	(.86)	(.12)	(− .03)	(− .18)
HrQOL: social/family well-being	CBCS		.30	.03	.25	.34
	SC		− .10	− .16	.32	.38
	(BGD ES^b^)	(− .04)	(.30)	(.17)	(− .20)	(− .08)
HrQOL: functional well-being	CBCS		− .27	.05	− .18	− .28
	SC		.66	.51	− .12	− .55
	(BGD ES^b^)	(.16)	(1.16)	(.75)	(.24)	(− .10)
Perceived stress	CBCS		− .53	− .06	− .23	− .59
	SC		.30	.12	− .30	− .54
	(BGD ES^b^)	(− .19)	(− .90)	(− .37)	(− .06)	(− .06)
Anxiety	CBCS		− .43	− .46	− .26	− .55
	SC		− .18	.01	− .28	− .37
	(BGD ES^b^)	(.34)	(.22)	(− .10)	(.55)	(.30)
Depression	CBCS		− .28	− .02	− .26	− .55
	SC		.14	.20	− .18	− .05
	(BGD ES^b^)	(.27)	(− .14)	(.02)	(.25)	(− .26)
Sleep disturbance	CBCS		− .51	− .48	− .66	− .53
	SC		− .28	.76	.92	− .03
	(BGD ES^b^)	(.77)	(.44)	(− .46)	(− .81)	(.13)
Fatigue	CBCS		− .14	− .12	− .27	− .52
	SC		.23	− .08	− .34	− .65
	(BGD ES^b^)	(− .27)	(− .66)	(− .36)	(− .23)	(− .24)
Sleep-related impairment	CBCS		− .25	− .07	− .18	− .48
	SC		.55	− .10	− .10	− .44
	(BGD ES^b^)	(− .41)	(1.36)	(− .45)	(− .55)	(− .66)
Anger	CBCS		− .48	− .25	.00	− .46
	SC		.11	.36	.08	.17
	(BGD ES^b^)	(.14)	(− .37)	(− .46)	(.09)	(− .55)
Pain interference	CBCS		− .37	− .15	− .02	− .14
	SC		− .14	− .12	− .67	− .73
	(BGD ES^b^)	(− .53)	(− .70)	(− .54)	(.10)	(.02)
Pain intensity	CBCS		− .64	− .15	.14	− .05
	SC		− .13	− .35	− .85	− .86
	(BGD ES^b^)	(− .15)	(− .66)	(.03)	(.82)	(.62)
MOCS: relaxation	CBCS			1.06	1.53	1.33
	SC			− .08	.43	.35
	(BGD ES^b^)	(.06)		(1.08)	(.88)	(.55)
MOCS total	CBCS			.49	1.01	1.30
	SC			− .04	.43	.44
	(BGD ES^b^)	(− .13)		(.33)	(.47)	(.48)
MOCS: assertiveness	CBCS			.00	.33	1.14
	SC			.03	.10	− .05
	(BGD ES^b^)	(− .22)		(− .29)	(− .03)	(.66)
MOCS: coping confidence	CBCS			.08	.70	.99
	SC			.07	.43	.52
	(BGD ES^b^)	(.07)		(.05)	(.12)	(.13)
MOCS: awareness of tension	CBCS			.44	.83	.59
	SC			− .18	.15	.54
	(BGD ES^b^)	(− .29)		(.33)	(.52)	(− .27)
Tx self-efficacy: coping with Psych.	CBCS			.14		
	SC			− .45		
	(BGD ES^b^)	(− .31)		(.31)		
Tx self-efficacy: coping with physical	CBCS			− .19		
	SC			.22		
	(BGD ES^b^)	(.61)		(.29)		
Tx self-efficacy: patient Comm.	CBCS			− .52		
	SC			.29		
	(BGD ES^b^)	(.22)		(− .60)		

^a^WGC ES is the change over time in the same condition, from T1 to T2, T1 to T3, T1 to T4, and T1 to T5

^b^BGD ES is the difference between the two conditions at each time point: T1, T2, T3, T4, and T5

### Change from baseline (T1) to start of HCV treatment (T2) to explore potential benefits after participation in four CBCS-HCV sessions

As shown in Tables 3 and 4, the CBCS-HCV condition showed potentially clinically significant improvements (i.e., ES > .35) in a variety of PROs, relative to gains made in the SC condition.

#### HRQOL and perceived stress

After four CBCS sessions (T1–T2), CBCS participants had moderate sized ES improvements in overall HRQOL on the FACT-GP (.38) and physical well-being (.44), both of which appeared to represent large ES differences between the two conditions at T2 (.86, .90). The CBCS group also demonstrated moderate ES improvements in perceived stress levels (.53), and this represented a large ES difference between the two conditions at T2 (.90).

#### Physical and mental symptoms

With regard to changes in baseline symptoms, the CBCS condition showed moderate ES benefits in several symptoms after only four CBCS sessions, including improvements in anger (.48), anxiety (.43), sleep disturbance (.51) pain intensity (.64), and pain interference (.37). The ES differences between the two conditions at T2 were in the small to moderate ES range for anger (.37), anxiety (.22), and sleep disturbance (.44) and in the moderate to large range for pain intensity (.66) and pain interference (.70). Even though improvements in depression, sleep-related impairment, and fatigue for the CBCS group were small over time (.25, .23, .28), these improvements suggested trends for the CBCS group to improve after only four CBCS sessions, while these symptoms all worsened during this timeframe in the SC group. The BGD at T2 was very large (1.35) for sleep impairment and moderate-to-large for fatigue (.66).

### Change from baseline (T1) to 1 month post-CBCS/post-HCV treatment (T5) to explore potential sustainable benefits of CBCS intervention after HCV treatment

As shown in Tables 3 and 4, the CBCS-HCV condition showed potentially significant improvements (i.e., ES > .35) in a variety of outcome measures from baseline (T1) to 1 month post-CBCS/post-treatment (T5).

#### HRQOL and perceived stress

In the CBCS group, all HRQOL (FACT-GP) scores improved from baseline to post-CBCS/post-HCV treatment, with moderate ES improvements in overall HRQOL (.51) and physical well-being (.59). However, HRQOL in the SC condition also improved from baseline to 1 month post-treatment. At 1 month post-CBCS/post-treatment, there were no significant differences in HRQOL between the two conditions on any HRQOL subscale. The CBCS group also demonstrated a moderate improvement in perceived stress levels (.59), but so did the SC condition (.54), and there was no difference between the two conditions post-CBCS/post-treatment (.06).

#### Physical and mental symptoms

With regard to changes in symptoms from T1 baseline to 1 month post-CBCS/post-treatment, the CBCS condition showed moderate benefits on most PROMIS® measures, including improvements in depression (.55), anger (.46), anxiety (.55), fatigue (.52), sleep disturbance (.53), and sleep-related impairment (.48). No significant sustainable improvements were found for pain intensity and pain interference in the CBCS condition. The SC condition did not show any improvements in depression, anger, or sleep disturbance but did show improvements in fatigue and pain intensity 1 month post-treatment. The BGDs at 1 month post-treatment were overall in the moderate range (.26–.66), with CBCS patients reporting lower depression, anger, fatigue, and sleep-related impairment scores, compared to patients in the SC condition.

#### Medication adherence and viral cure

According to the objective pill count data averaged across all available treatment weeks for both conditions, there was no difference between the groups in medication adherence. The proportion of ideal versus actual doses taken by CBCS participants was 96% (84–100%) for all medications (i.e., SIM, SOF, RBV). One patient in the CBCS condition accidentally double-dosed sofosbuvir for 1 week bringing down the group average. The proportion of ideal versus actual doses taken by SC participants was 98% (91–100%). According to the 7-day self-reported recall data averaged across all treatment weeks, the proportion of doses taken was 99.9% for the CBCS and 99.6% for SC. With regard to viral cure, 100% of patients in both conditions achieved undetectable HCV RNA at 4 or 12 weeks post-HCV treatment, suggesting all had achieved viral cure.

### Potential mechanisms

#### Active cognitive behavioral ingredients

Tables 3 and 4 display the means and ESs over time from the MOCS survey which measures patient-reported cognitive behavioral skill acquisition that the intervention targeted (i.e., relaxation, assertiveness, awareness of tension, confidence in coping). CBCS participants reported a gradual increase in utilization of all cognitive behavioral skills over time, with moderate improvements after four CBCS sessions (.49) and extremely large improvements 1 month post-CBCS (1.30). Improvements in CBCS participants’ use of relaxation skills increased over time with extremely large improvements (1.56, 1.33), and the BGDs of these skills at each time point ranged from moderate to extremely large. The use of assertiveness skills increased over time in the CBCS condition, with an extremely large WGC from baseline to 1 month post-CBCS (1.14) and a moderately large BGD (.66) compared to SC at this 1-month post-CBCS time frame. Awareness of tension increased in the CBCS condition over time, peaking at the end of the CBCS intervention with a large ES (.83) and maintaining this skill at 1 month post-CBCS. Confidence in ability to cope increased over time in both groups with large ES changes over time for the CBCS condition (.99) and moderate ES changes in the SC condition.

#### Self-efficacy

Table 3 displays the means from *The Hep C Treatment Self-Efficacy Survey* at T1 and T3 post-CBCS intervention and ESs for three subscales (i.e., confidence in patient communication, coping with physical symptoms, coping with psychological symptoms). Higher scores indicate higher patient-reported self-efficacy. In both conditions, all treatment self-efficacy scores stayed the same or decreased from T1 to T3.

#### Group therapeutic processes

Nonspecific therapeutic factors of group interventions (e.g., empathy, group cohesiveness) may positively impact key outcomes. On average, participants reported that the patient-therapist bond was very positive (*M* = 6.26, SD = .70) and participants experienced a great deal of group cohesiveness (*M* = 6.33, SD = .71). Participants also reported that their group experience was extremely favorable (*M* = 6.29, SD = .71).

### Study feasibility measures

**Fig. 9:**
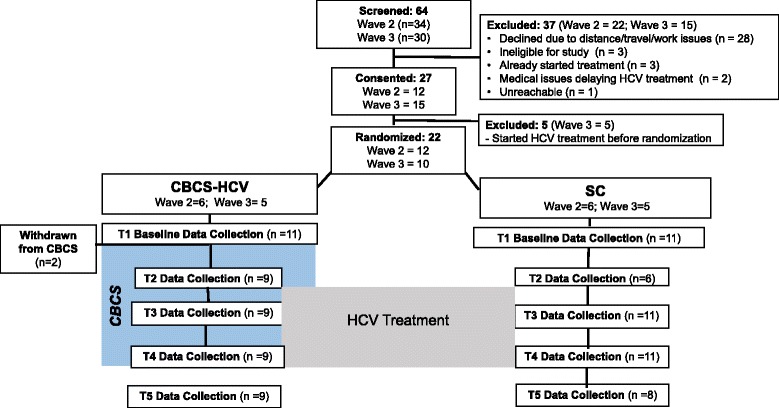
Study flowchart. Note: two participants withdrew after randomization and baseline due to transportation issues and delays in treatment. Period of time in which both the CBCS-HCV and SC groups received HCV treatment.  Period of time in which only the CBCS-HCV received the CBCS intervention.  Intervention group.  Standard of care group.  Measurement time points

The study flowchart is displayed in Fig. 9.

#### Feasibility of recruitment, enrollment, and randomization

As shown in Fig. 9, the medical records of 64 patients were reviewed as initial screening process for both waves 2 and 3. Potentially eligible patients were contacted via telephone. Of 64 screened patients, 27 (42%) consented to participate in the study, and 22 of the 27 who were consented (81%) were randomized. The primary reasons for screen failures were patient barriers to attending in-person CBCS sessions: proximity to the center, transportation issues, and work conflicts. Recruitment for wave 2 took approximately 30 days, and recruitment for wave 3 took approximately 64 days. Due to prescription refill requirements, the clinical staff required patients to initiate HCV treatment within 1–2 weeks of home delivery or else risk having future refills expire. Thus, we were challenged with retaining consented patients who had obtained their medications and were in the queue for randomization, while concurrently trying to rapidly recruit new patients. We were able to successfully complete block randomization of 12 patients as the protocol dictated during wave 2 despite these clinical constraints. However, we decided to conduct block randomization for wave 3 when we had 10, not 12, consented patients. Five out of 27 patients who were consented started their HCV treatment before block randomization could be conducted, leaving 22 to randomize.

#### Feasibility of study retention

Of the 22 patients randomized, two patients randomized to CBCS-HCV and who completed baseline T1 data were withdrawn before the CBCS-HCV intervention began (see Fig. 1). Therefore, 20 patients (91%) were retained and completed the full study.

#### Feasibility of delivery and retention in CBCS-HCV group intervention

All nine (100%) patients who started the CBCS-HCV intervention were retained in the study, and none were lost to follow-up. Attendance at all nine CBCS-HCV sessions was exceptional; only one patient missed the first session; otherwise, attendance was 100% at every session. High attendance at these sessions is a strong indicator of patient enthusiasm and satisfaction with the CBCS-HCV intervention. There were no patient-reported harms or unintended effects of the study in either group.

#### Feasibility of PROM data collection and missing data

The overall rate of data completion for key PROMs via paper and pencil administration was 89% throughout the study. Data collection at T1 was 100%. At T2, 75% of PROMs were collected. Data missed at T2 were all from the SC group who did not have regular clinic visits scheduled and who did not return surveys sent via post mail. Also at T2, four participants (two CBCS, two SC) completed survey packets after starting HCV treatment (1–18 days into treatment). A comparison of PRO data collected during versus before treatment revealed no trends for outlying data (i.e., the data were in a similar range for both groups). At T3, 97% of PRO data were collected. At T4, 95% of PRO data were collected. At T5, 80% of PRO data were collected with missing data due to nonattendance at 1-month clinic visits and not returning surveys via post mail. Finally, we observed incomplete, poor quality data on four out of 18 observer-rated therapist protocol fidelity forms (nine sessions × two waves), which we were unable to include in the analysis of protocol fidelity.

#### Protocol fidelity

According to the protocol, an exclusion criterion was being prescribed 24 weeks of treatment. However, two patients in the SC condition were found to be infected with HCV genotype 3 which required extension of treatment from 12 to 24 weeks. Both participants remained in the study, and the evaluation of T5 PRO data from their treatment week 16 visit revealed no concern for outlying data (i.e., within the range of other scores). Overall therapist fidelity was 85% in wave 2 and 92% in wave 3 on the observer-rated protocol fidelity forms completed after each session. The average observer rating of the therapist’s competency to conduct each of the nine modules was 3.9 on a scale ranging from 0 to 4. The therapist’s competencies that were consistently rated a four out of four across all nine modules included warmth, genuineness, showing interest, and empathy.

#### Patient acceptability

Based on patient report at the end of each of the nine modules, the average rating across all nine sessions and all patients was 4.51 on a 5-point scale (SD = 0.18), indicating a high level of satisfaction with the content and group dynamics. Participants consistently reported that they had a good session (*M* = 4.63, SD = .20), group members seemed to genuinely care about each other (*M* = 4.46, SD = .31), and they intended to remain in the program (*M* = 4.87, SD = .11). However, participants also indicated there was not enough time for discussion and review (*M* = 3.76, SD = .57) and there was too much information to cover (*M* = 3.92, SD = .62) during the sessions.

Examples of participants’ written feedback included “I always enjoy every session,” “I loved all the sessions and feel they were most beneficial to my day to day living; wonderful group and facilitators”, “It was good to have people I could relate to in my group; I have learned to relax and discuss my problems,” “Affirmation self-talk; I will definitely practice this,” “Learning about anger; I learned ways to help control anger using a control plan,” “Relaxation, learning about imagery, group discussion,” “I can really use what I learned today to access and positively modify my thoughts,” “Beginning to recognize what stresses me,” and “I liked learning how to relax and what things were stressors and how I act to those stressors.”

## Discussion

The CBCS-HCV pilot and feasibility study findings presented here extend our previous work with the initial wave 1 of study participants [12]. Similar to the results of wave 1, many aspects of the study design, intervention implementation, and collection of PROMs were feasible. The group intervention and modules were highly acceptable to patients. We also identified some specific challenges to address in future CBCS-HCV studies to improve study design, implementation, and PROM data collection, in order to conduct a successful efficacy trial. Importantly, the findings from this pilot study will aid in the selection of key PROs to evaluate in subsequent studies.

The CBCS-HCV intervention appeared to positively impact several, though not all, of the PROs, with many effect size estimates suggestive of clinical benefits. Specifically, patients’ perceptions of stress and some components of HRQOL appeared to be positively affected by the intervention. Moreover, mental health symptoms of depression, anger, and anxiety showed dramatic improvements that were sustained 1 month after HCV treatment and the intervention were completed, compared to the SC condition. There were also sustained improvements following the CBCS-HCV intervention in sleep disturbance and sleep-related impairment, but not for pain intensity and interference. These preliminary data suggest which PROs may or may not be impacted by the CBCS-HCV and thus which PROs are worth investigating in future studies of the CBCS-HCV.

Per Wilson and Cleary, HRQOL is a broad, multi-faceted construct affected by many confounding variables [16]. We used the FACT-GP PROM to evaluate HRQOL and found that the total score and the FACT-GP physical well-being score improved initially after the first four CBCS modules, which may have been due to intervention components enhancing lifestyle behaviors, such as nutrition, hydration, physical activity, and sleep hygiene. These improvements in overall HRQOL and physical well-being were sustained at 1 month post-treatment/post-intervention. In contrast, the intervention was not intended to directly target family/social functioning, and thus, it is not surprising that this subscale score did not change over time. Emotional and functional well-being were addressed by the intervention, but the FACT-GP subscale score did not improve substantially over time or no differences between the two conditions were observed on these subscales. This is inconsistent with the significant improvements we observed on the PROMIS® depression, anxiety, and anger PROMs. It may be that the FACT-GP PROM is too non-specific or multidimensional to capture precise changes over time and perhaps the PROMIS® short forms are more sensitive to change.

The Perceived Stress Scale scores improved dramatically during the CBCS-HCV intervention while stress levels worsened in the SC condition. CBCS-HCV benefits were sustained 1 month later; however, perceived stress levels in the SC group also improved after treatment ended. Stress reduction in the SC condition could have been due to completion of antiviral therapy, being cured from HCV, or both. The CBCS-HCV provides substantial training in stress reduction techniques, including starting each session with a new relaxation exercise, and this may account for the clinical benefits seen early in the CBCS-HCV group. With many HCV patients having co-occurring mental health, psychosocial instability, and addiction issues, reductions in psychological stress may be an important health outcome to target in this population. The Perceived Stress Scale appears to be an appropriate measure to capture patient-reported stress improvements and could potentially be complemented in future studies by objective measures of physiological stress (e.g., cortisol, immune functioning) [28–30].

The prevalence of mood disorders in the HCV population (11–68%) is much higher compared to the general population (5–12%), particularly for major depression (24–68%) [31–33]. Our findings suggest that mood disturbance may improve meaningfully as a result of the CBCS-HCV intervention. Patient-reported depression, anger, and anxiety improved after four CBCS-HCV sessions, whereas these psychological PROs did not change in the standard of care condition. Improvements were maintained 1 month post-intervention in the CBCS group, whereas depression and anger worsened or stayed the same in the SC condition. Modules that specifically target stress reduction, relaxation, and negative thinking may be responsible for improvements in these PROs and represent important targets for future studies. The NIH PROMIS® measures may be particularly incisive at capturing mental and physical symptoms.

Sleep disturbance is a common complaint among patients with HCV and can affect mental and physical health [34]. One module was devoted to sleep hygiene techniques, although sleep was discussed in several subsequent sessions. We found that sleep disturbance improved in the CBCS-HCV condition relative to the SC condition after four CBCS sessions and these benefits were maintained 1 month post-intervention. No improvements were observed in the SC group. Sleep-related impairment also improved over time in the CBCS group, with improvements maintained 1 month post-intervention. Fatigue is one of the most common features associated with HCV and, therefore, was addressed via multiple cognitive and behavioral strategies (e.g., relaxation, sleep hygiene, activity-rest cycles, pacing) [35]. The CBCS group showed improvements in fatigue at 1 month post-intervention; however, the SC condition also reported less fatigue post-treatment compared to baseline so that no BGD was observed. It is difficult to discern the beneficial effects of the intervention versus viral cure on the 1-month outcomes like fatigue; however, given that fatigue is such a salient symptom associated with HCV, it will remain important to address fatigue symptom improvements in a future efficacy study and evaluate this important PRO. More time may need to be devoted to practicing healthy sleep hygiene skills to augment these modest improvements.

The CBCS-HCV intervention did not produce any lasting effects on pain outcomes as measured by the PROMIS® Pain Intensity and Pain Interference surveys. While initial reductions were reported, these improvements were not maintained at 1 month post-intervention, and the SC condition reported less pain at follow-up. Some CBCS-HCV intervention modules could have indirectly improved coping with pain (e.g., relaxation exercises, activity-rest cycling, cognitive strategies for negative thoughts), but the “dose” may have been insufficient to create lasting changes. Moving forward with the CBCS-HCV program, it is unlikely that pain will be impacted substantially without adding more pain management training.

Previous studies suggest that HCV patients have worse mental and physical health compared to the US general population [4, 36]. Consistent with this literature, our sample reported worse baseline scores on a majority of PROMIS® measures relative to the general US population (*T*-score = 50) on which the scores were standardized. However, by 1 month post-intervention, several PROMIS® scores improved or fell below the population *T*-score. Thus, psychosocial interventions like the CBCS-HCV may aid in normalizing HCV health outcomes to be commensurate with the health of the larger US population.

This pilot study sheds light on potential mechanisms, or mediators of change, that may be partially responsible for clinical benefits observed in outcomes. PROMs were the only viable means of capturing subjective patient experiences of mediators that may underlie improvements. Several specific cognitive behavioral strategies (e.g., relaxation) increased over time and may be correlated with and underlie clinical improvements [37]. Secondly, nonspecific therapeutic processes that were rated as high (e.g., group cohesion, acceptance) may also be responsible for improvements and warrants future examination [38]. In contrast, the pilot data do not suggest that HCV treatment self-efficacy improved over time and thus is unlikely to be responsible for clinical improvements. The CBCS-HCV intervention was not specifically designed to target the four foundations of self-efficacy [39], which might explain the lack of change seen on this scale. In a future efficacy study, a secondary analysis of changes in mechanisms (i.e., mediating variables) correlated with changes in outcome variables would provide insight into the specific cognitive behavioral, lifestyle, and group processes that facilitate clinical improvements.

With regard to study feasibility, we identified some challenges, all of which can be addressed in future CBCS-HCV studies. The greatest challenge was recruiting patients in rapid succession in order to conduct block randomization. Another challenge was transportation issues including long travel distances that precluded some patients from attending in-person intervention sessions and thus they declined to participate. Thirdly, while PRO data collection was satisfactory (89%), we identified issues to address in future studies to enhance PRO completion rates. These issues can be mitigated by improving coordinator training, real-time quality assurance checks, and using phone-based or electronic data capture systems, such as REDCap (https://www.project-redcap.org/). Paper and pencil administration of PROMs can be fraught with human error if not monitored in real-time. Thus, future studies will transition to PRO collection via phone-based surveys or REDCap, which have proven successful in other studies [40]. Finally, participants recommended expanding the number of sessions to allow for more practice, discussion time, and group bonding. A strength of the study was near-perfect retention in the CBCS-HCV groups. Strategies that may have increased retention included positive group dynamics and peer support and phone reminder calls from the research coordinators regarding date, time, and location of the next group. These experiences and lessons learned from pilot testing were tremendously useful and will strengthen future CBCS-HCV trials and intervention implementation.

Given what we have learned from conducting these pilot studies, changes to some of the study design features and the way in which the CBCS-HCV group intervention is delivered will be needed. For example, it may be useful to consider offering the CBCS-HCV intervention as an alternative healthcare service to all people who have been infected with chronic HCV, not just those embarking on HCV treatment. With prevalence of mental health and substance use issues, stress management modules could address underlying antecedents to maladaptive cognitions and behaviors. Likewise, lifestyle modifications (e.g., changes to eating and activity behaviors) may improve overall liver health for patients at risk for other liver diseases, such as those with fatty liver disease. From a study design perspective, expanding eligibility criteria to all patients with HCV would mitigate the challenges encountered when recruiting only a subgroup initiating HCV therapy.

A second consideration involves delivering the CBCS-HCV group intervention using telehealth videoconferencing technology, as opposed to in-person groups. Travel distance and transportation issues are prevalent patient-level barriers that stymie access to innovative healthcare services and research studies, particularly in rural states where patients live long distances from academic medical centers. Delivery of interventions via telehealth videoconferencing technology may be a cutting-edge alternative to in-person delivery, supported by a growing body of evidence. Videoconferencing is an efficacious mode of delivering treatment for a wide range of mental and physical conditions, and a variety of different interventions have been delivered including cognitive behavioral, supportive, and educational [41–45]. Reducing geographical and financial barriers may lead to higher rates of study enrollment, intervention reach, and clinical effectiveness. Thus, telehealth platforms like videoconferencing may increase the reach, uptake, and dissemination of useful interventions to enhance the overall health of the HCV population [46].

## Conclusions

In conclusion, we have completed two pilot feasibility studies with three waves of participants and examined various elements of feasibility, patient acceptability, and effect size estimates of clinical improvements in PROs that may be achieved with the CBCS-HCV [12]. Patient enthusiasm for the intervention combined with findings that are suggestive of clinical benefits in psychological stress, depression, anxiety, anger, fatigue, and sleep all provide evidence to support further evaluation of the CBCS-HCV. Several PROMs, namely the PROMIS® short forms, the Perceived Stress Scale, and the MOCS, appear sensitive enough to capture diverse and precise improvements in patient outcomes. We conclude that expanding the eligibility criteria to include all people who have been infected with HCV and delivering the CBCS-HCV via videoconferencing telehealth technology would not only improve future trials, but more importantly, could expand the dissemination and implementation of this potentially useful psychosocial intervention for the HCV patient population.

## Abbreviations

BGD: Between-group difference
CBCS-HCV: Cognitive-Behavioral Coping Skills group intervention for HCV
DAA: Direct-acting antiviral
ES: Effect size
FACT-GP: Functional Assessment of Cancer Therapy-General Population
HCV: Hepatitis C virus
HrQOL: Health-related quality of life
MOCS: Measure of Current Status
NIH: National Institutes of Health
PROMIS®: Patient-Reported Outcomes Measurement Information System®
PSS: Perceived Stress Scale
RCT: Randomized controlled trial
SC: Standard of care
SD: Standard deviation
WGC: Within-group change
